# Faunal Responses to Habitat Disturbance: Do the Principles Explaining Responses of Ant Communities Also Apply to Terrestrial Reptiles?

**DOI:** 10.1002/ece3.70939

**Published:** 2025-03-06

**Authors:** Angga Rachmansah, Keith Christian, Brett P. Murphy, Christine Schlesinger, Alan N. Andersen

**Affiliations:** ^1^ Research Institute for the Environment and Livelihoods Charles Darwin University Brinkin Northern Territory Australia

**Keywords:** disturbance, ectotherm, functional composition, habitat openness, terrestrial reptile

## Abstract

Disturbance is fundamental to the state and dynamics of biological communities, and understanding biotic responses to disturbance is critical to effective biodiversity conservation. However, a predictive understanding of how faunal communities respond to habitat disturbance remains elusive. Recently, a conceptual framework centred on habitat openness was developed for understanding ant responses to disturbance. It proposes that habitat openness is a fundamental driver of variation among ant communities, and that the primary impacts of disturbance are mediated through ant functional responses to changes in openness. Like ants, terrestrial reptiles are ectotherms and are therefore especially sensitive to disturbance‐induced increases in habitat openness because of changes in the thermal environment. Therefore, reptiles might also be expected to conform to a disturbance framework based on habitat openness. Here we assess the extent to which this occurs by combining a quantitative analysis of recent publications with a broader synthesis of the literature. We found strong support for the framework applying to terrestrial reptiles. We suggest that the framework can be strengthened by a mechanistic understanding of functional traits in relation to habitat openness. For ectotherms, ecophysiological traits could be particularly important for responding to disturbance‐mediated changes in microclimate, but habitat openness also influences other important factors such as food availability and predation. Finally, the framework appears to be highly applicable to a wider range of faunal groups beyond ants and reptiles.

## Graphical abstract

Understanding biotic responses to disturbance and their underlying mechanisms is critical to effective biodiversity conservation, but a predictive understanding of how faunal communities respond to disturbance remains elusive. In this paper, we review the applicability of a disturbance framework centred on habitat openness to understand terrestrial reptile responses to habitat disturbance. We find that all five principles of the framework strongly apply to terrestrial reptiles and that the framework may also be relevant to animals more generally.

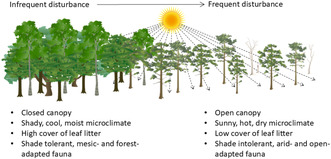

## 1 Introduction

**FIGURE 1:**
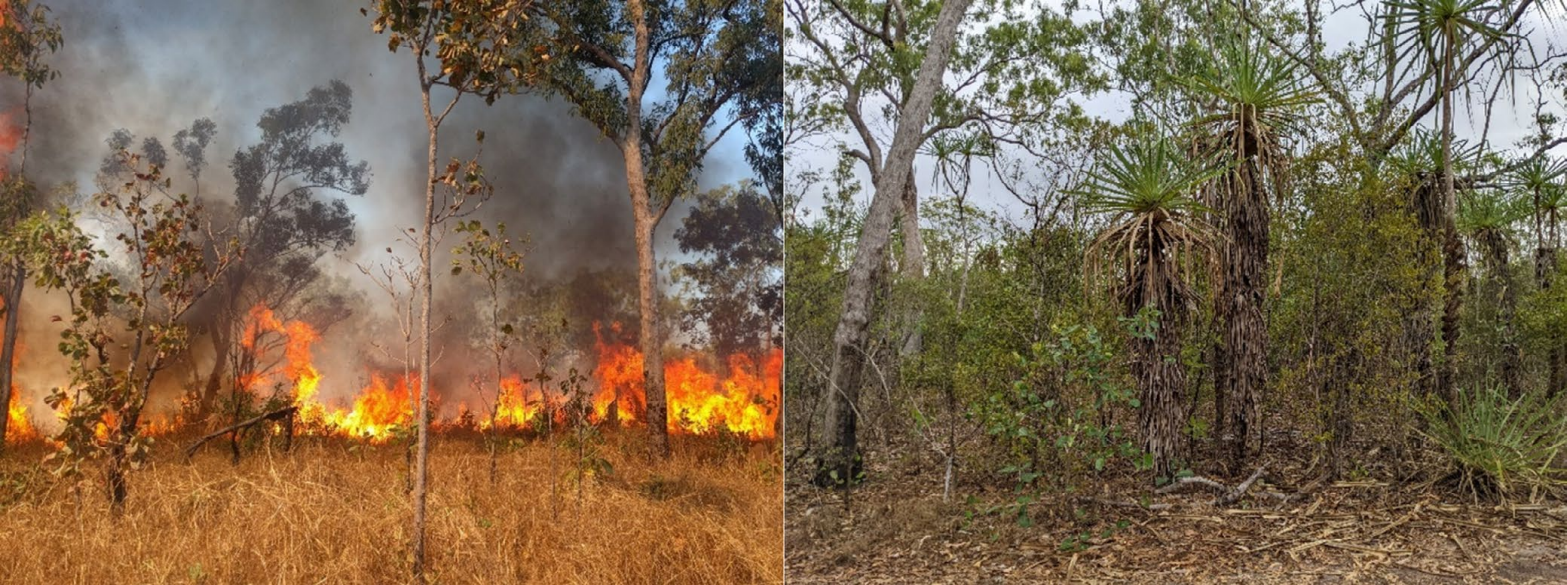
Fire maintains the open vegetation structure characteristic of tropical savannas (left), which in mesic environments would otherwise transform into a closed‐canopy ecosystem (right).

Disturbances such as fire, grazing, floods and storms are integral features of natural ecosystems, and play a key role in the structure, composition and function of biological communities. In the tropics, for example, disturbance helps maintain open, grass‐dominated ecosystems in regions of high but highly seasonal rainfall, by preventing their transformation into closed‐canopy forests (Figure 1; Bond, Woodward, and Midgley 2005; Dantas et al. 2016; Hoffmann et al. 2012; Murphy and Bowman 2012). These grassy ecosystems are dominated by shade‐intolerant species whose diversity is often comparable to that of tropical forests (Murphy, Andersen, and Parr 2016). More generally, disturbance plays a key role in maintaining biodiversity by creating and maintaining habitat heterogeneity, allowing the coexistence of species with different ecological requirements (Connell 1978; He, Lamont, and Pausas 2019; Huston 1979; Petraitis, Latham, and Niesenbaum 1989). An understanding of disturbance is not only essential for understanding community dynamics, but also provides the foundation for effective conservation management.

Following Andersen (2019), we use Grime's (1977) definition of disturbance as any factor that removes biomass, focusing on the effects of habitat disturbance through the removal of vegetation biomass. Predicting the impacts of habitat disturbance on biodiversity requires an understanding of the mechanisms through which these impacts occur. Habitat disturbance can influence fauna either directly, such as through mortality caused by flames or smoke, or indirectly through short‐ or long‐term habitat modification (Andersen 2019). The relative importance of these effects depends on the species concerned and the pre‐disturbance state of vegetation, along with the type, severity and frequency of disturbance (White and Pickett 1985). Most ecosystems and their constituent species have evolved with natural disturbance regimes, making them resilient to at least some forms of disturbance (Johnstone et al. 2016). However, biodiversity responses to disturbance are often so highly variable that they seem too complex to predict (Lindenmayer et al. 2008; Nimmo et al. 2014; Pulsford, Lindenmayer, and Driscoll 2016). Moreover, disturbance regimes have changed dramatically during the Anthropocene, and predicting responses to new disturbance regimes is even more challenging.

**FIGURE 2:**
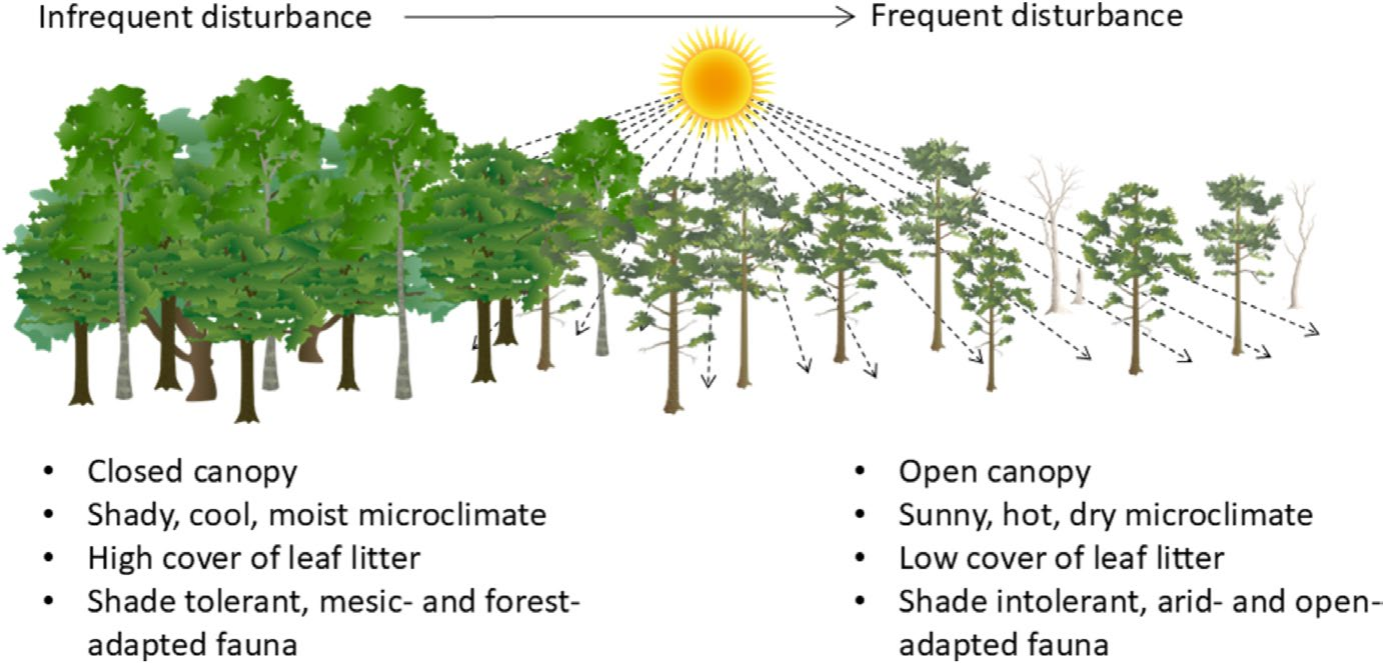
Schematic overview of the importance of habitat openness in Andersen's (2019) disturbance framework. Drawn using icons from Integration and Application Network (ian.umces.edu/media‐library).

Recently, Andersen (2019) introduced a conceptual framework for predicting ant responses to disturbance, and suggested this was likely to have broader applicability to other faunal groups. The framework applies to moderate levels of disturbance such as fire, grazing and selective logging, rather than to land conversion, and identifies habitat openness as a key mechanism for mediating ant responses, especially through its influence on microclimate (Figure 2). The framework comprises five principles: (1) The most important effects of habitat disturbance are typically indirect, via habitat structure, microclimate, resource availability and competitive interactions; (2) Habitat openness is a key driver of variation in ant communities; (3) Ant community responses to disturbance are to a large degree determined by their responses to habitat openness; (4) The same disturbance will have different effects on ants in different habitats, because of different impacts on habitat openness; (5) Ant community responses to the same disturbance will vary according to ant functional composition and biogeographic history in relation to habitat openness.

In this synthesis paper, we assess the extent to which Andersen's (2019) disturbance framework applies to terrestrial reptiles. Like ants, reptiles are a highly diverse and ecologically important faunal group in most terrestrial ecosystems, especially in warmer regions (Roll et al. 2017). Additionally, like ants, reptiles are ectotherms whose physiological function depends on the ambient thermal environment and are therefore highly sensitive to changes in it (Angilletta, Niewiarowski, and Navas 2002; Nowakowski et al. 2018; Ramalho et al. 2023; Taylor et al. 2021). Terrestrial reptiles can therefore be expected to be highly responsive to variation in microclimate mediated by habitat openness (Principle 2), and this is indeed the case. By determining levels of direct insolation and therefore opportunities for thermoregulation, habitat openness affects many aspects of reptile biology (Ferreira, Žagar, and Santos 2017; Sillero et al. 2009), including rates of foraging (Fouts et al. 2017; Sartorius, Vitt, and Colli 1999) and reproductive success (Markle et al. 2021; Shine, Barrott, and Elphick 2002). In addition to microclimate, variation in habitat openness has important implications for food availability and predation risk (Daly, Dickman, and Crowther 2008). This is reflected in functional morphology (Calsbeek, Knouft, and Smith 2006; Schneider et al. 1999); for example, lizards in open habitats tend to have long limbs for fast locomotion (Goodman 2009; Melville and Swain 2000). Given the above, it is not surprising that variation in habitat openness is a primary factor influencing reptile diversity and distribution (Balaji, Sreekar, and Rao 2014; Pike, Webb, and Shine 2011).

Given the well‐established relationship between habitat openness and variation in reptile communities, our review focuses on the four principles (1, 3, 4 and 5) that directly relate to disturbance. We first provide a structured analysis of the extent to which terrestrial reptiles conform with the principles through reviewing recently published papers on reptile responses to disturbance. We then integrate this analysis into a broader synthesis of the literature to discuss each of the four principles in turn.

## 2 Structured Review

**FIGURE 3:**
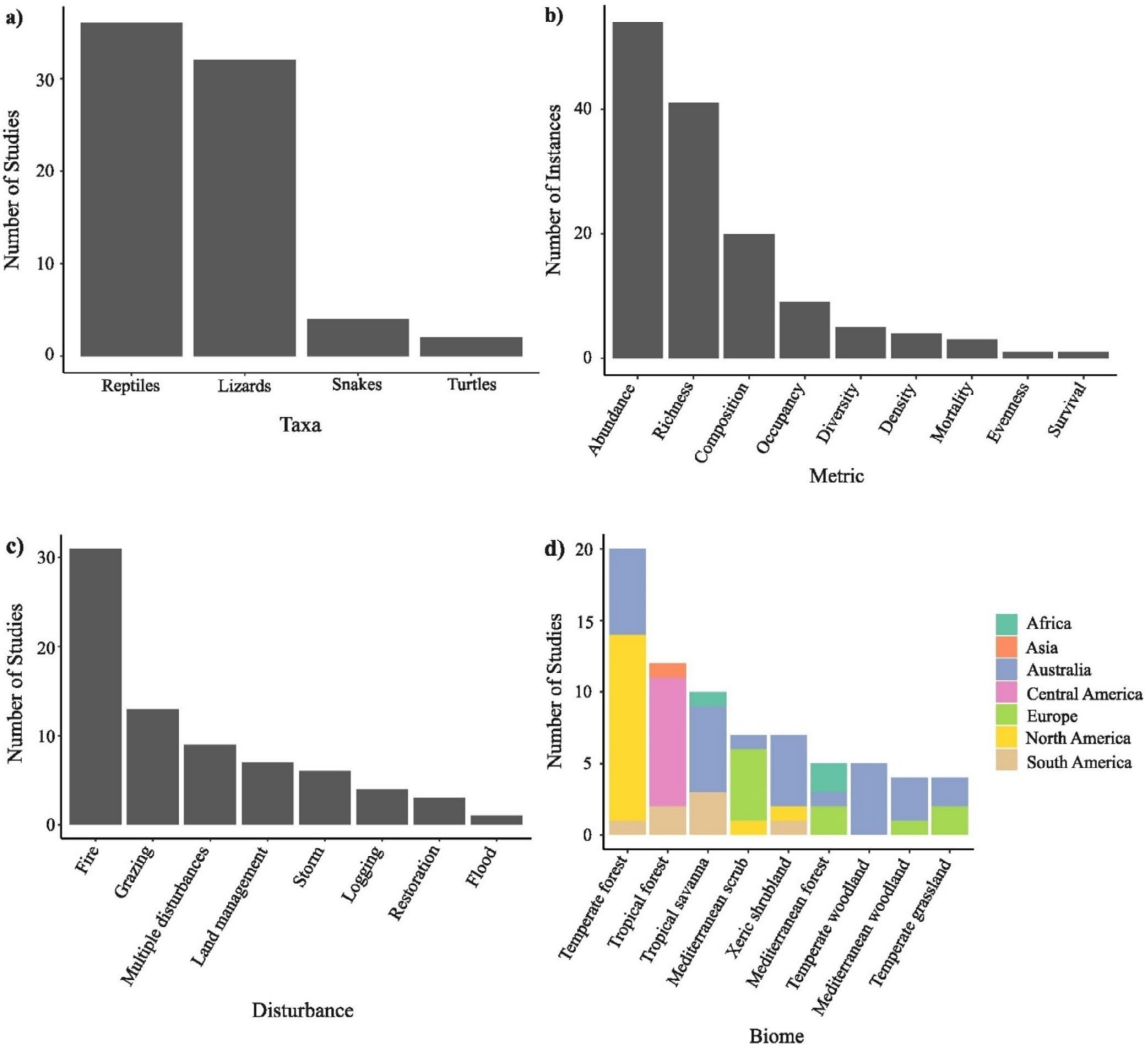
Summary of scope of studies of reptile responses to disturbance (and restoration following disturbance) obtained from a structured review of papers published from 2017 to 2022: (a) focal taxa, (b) metric, (c) disturbance agent and (d) biome. We followed a simplified biome classification of Olson et al. (2001).

**FIGURE A1:**
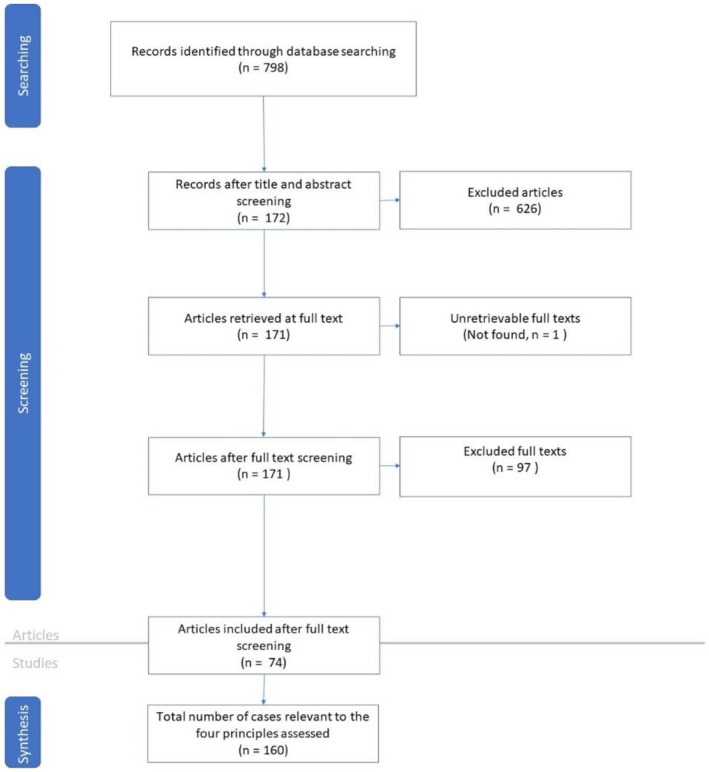
Modified ROSES flow diagram (Haddaway et al. 2017) of quantitative review of papers published on responses of terrestrial reptiles to disturbance from 2017 to 2022 inclusive (including early access).

Our structured review considered papers published between 2017 and 2022 (including early access in 2022) inclusive as indexed in the database of Web of Science Core Collection in August 2023. We used combinations of keywords “reptil*” OR “lizard*” AND “disturbance*” OR “fire*” OR “log*” OR “graz*” for the topic, which identified 796 papers. The results were then screened for those that (1) described an empirical study of the impacts of moderate levels of disturbance (i.e., not ecosystem conversion), and (2) measured species richness, diversity, composition, abundance, survival, evenness, mortality, density or occupancy. The resulting 171 papers then received a final screen for relevance to the four principles, giving a final 74 papers for analysis (Appendix 1: Figure A1). These 74 papers contained a total of 160 cases of direct relevance to one of the four principles (Appendix 2: Table A1), which we consider to be sufficient for a quantitative assessment of the extent of support for the principles. Approximately half of the papers considered a range of reptile groups, and most of the others considered lizards only (Figure 3). Most papers examined abundance, richness and/or composition as metrics, and fire or grazing as the disturbance agent. The studies were conducted in a wide range of biomes, but especially temperate forests and tropical forests and savannas (Figure 3).

## 3 Principle 1. The Most Important Effects of Habitat Disturbance Are Typically Indirect, Through Effects on Habitat Structure, Microclimate, Resource Availability and Competitive Interactions

**FIGURE 4:**
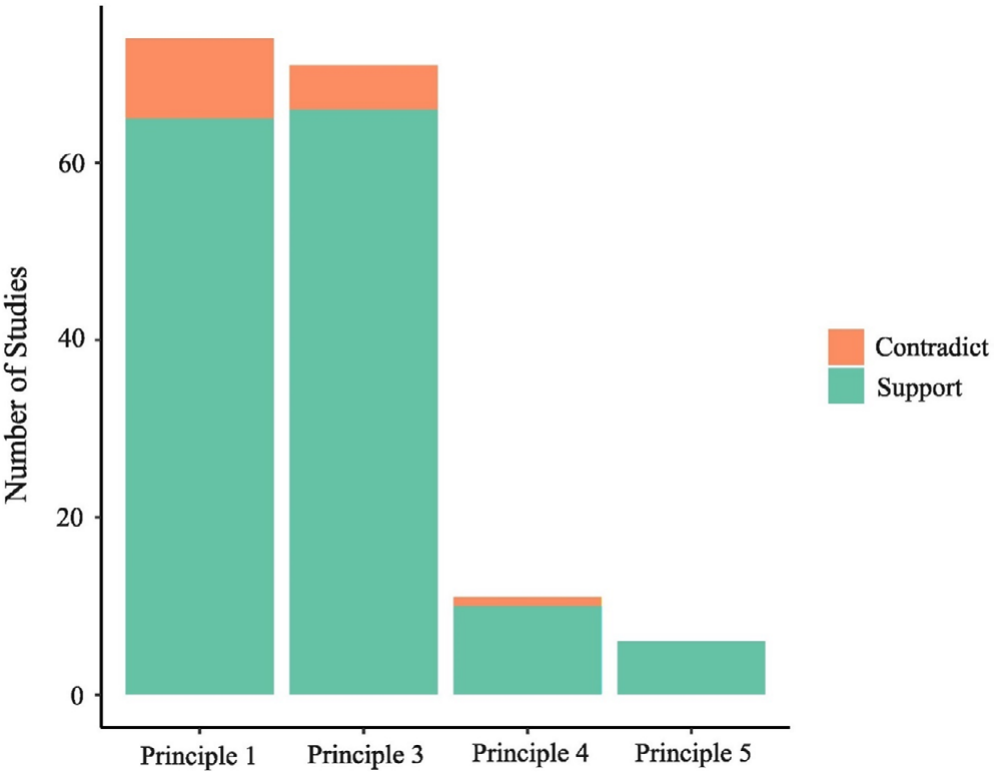
Number of studies from the structured review that support or contradict the principles of ant responses to disturbance.

The structured review provided very strong support (64 out of 74 instances) for this principle (Figure 4; Appendix 2: Table A1). Few studies directly measured mortality, although low levels of mortality were sometimes assumed based on in situ persistence soon after disturbance (e.g., Richter et al. 2021; Santos et al. 2022). High levels (up to 48%) of mortality were documented in a few instances, mostly involving fire and turtles (Oliveira et al. 2019; Buchanan, Steeves, and Karraker 2021; Heaton et al. 2022). Among terrestrial reptiles, direct mortality during fire is often particularly high in testudines (Platt, Liu, and Borg 2010), reflecting their limited mobility and hence capacity to find shelter (Heaton et al. 2022; Howey and Roosenburg 2013).

Unsurprisingly, reptile mortality rates during fire increase with increasing fire intensity (Griffiths and Christian 1996; Jolly et al. 2022; Tomas et al. 2021). For example, frilled‐neck lizards ( *Chlamydosaurus kingii* ) in Australian tropical savanna survive low‐intensity fires by remaining in the tree canopy, but experience high (ca. 30%) mortality during high‐intensity fires that scorch the canopy (Griffiths and Christian 1996). Mortality can be particularly high when high‐intensity fires coincide with peak reptile activity, such as during the breeding season (Jordaan et al. 2020; Platt, Liu, and Borg 2010). High‐intensity fires can even cause substantial mortality of burrowing species (Homan 2014; Smith et al. 2012), through asphyxiation, heat‐induced cardiac arrest or toxic gas inhalation (Jordaan et al. 2020). Species with slow life histories may be vulnerable to disturbance‐induced mortality, as are populations that are declining due to other factors (Ensbey et al. 2023).

Despite the potential for substantial direct mortality, there is often little or no mortality of reptiles during fire (Costa et al. 2013; Fenner and Bull 2007; Hellgren et al. 2010; Russell, Van Lear, and Guynn 1999), reflecting the availability of shelters such as burrows, hollow logs and termite mounds (Jolly et al. 2022; Nimmo et al. 2019, 2021). For example, lizards in a Brazilian savanna experienced little or no direct mortality from fire because they sought protection in burrows and termite nests (Costa et al. 2013). An absence of genetic bottlenecks in Portuguese wall lizards (*Podarcis guadarrame*) indicated high rates of survival in response to recurrent wildfires, most likely because they can shelter in crevices and burrows (Ferreira et al. 2019). Other studies in Australian tropical savanna (Nicholson, Lill, and Andersen 2006), South African tropical savanna (Jordaan et al. 2020, 2019), North American wetland (Schneider and Kashian 2014) and North American temperate mixed forest (Brown et al. 2014) found similar evidence of little or no direct mortality due to fire.

Many animals from fire‐prone environments possess morphological, physiological or behavioural traits that enable them to avoid direct mortality from fire (Pausas and Parr 2018). Some reptile species respond to smoke from nearby fire (Álvarez‐Ruiz, Belliure, and Pausas 2021; Mendyk, Weisse, and Fullerton 2020), potentially allowing them to move away or find shelter in situ. Mendyk, Weisse, and Fullerton (2020) found that captive shingleback lizards (*Tiliqua rugosa*) exposed to smoke from an accidental fire displayed increased tongue‐flicking and escape behaviours. Spanish populations of the Algerian sand racer (*Psammodromus algirus*) from fire‐prone habitats showed higher responsiveness to smoke compared to those from non‐fire‐prone habitats (Álvarez‐Ruiz, Belliure, and Pausas 2021). In addition to detecting smoke, some reptiles respond to the sound of approaching fire (Álvarez‐Ruiz et al. 2023).

The extent of change and recovery of key habitat features (such as microclimate, shelter, food) following a disturbance shape the trajectory of population persistence and recovery (Driscoll et al. 2012; Letnic et al. 2004; Lindenmayer et al. 2008). For example, the removal of vegetation cover by fires in Chile diminished microhabitat availability for the arboreal slender lizard ( *Liolaemus tenuis* ), leading to its extirpation in recently burned areas (Zúñiga 2020). The rapid recovery of vegetation post‐disturbance often mediates a swift recovery of reptile populations (Abom and Schwarzkopf 2016; Aviles‐Rodriguez, De León, and Revell 2023; Davis and Doherty 2015; Gorissen, Greenlees, and Shine 2018; Moreno‐Rueda et al. 2019; Richter et al. 2021). However, in some cases the recovery of reptiles following disturbance can be far slower, particularly for species reliant on specific habitat components, such as those exclusive to old‐growth forests (Hernández‐Ordóñez, Urbina‐Cardona, and Martínez‐Ramos 2015; Hu et al. 2013). Favourable habitat conditions following disturbance may even compensate for high mortality during it. For example, frilled‐neck lizards are most common under a regime of high‐intensity fires because high levels of mortality are more than offset by increased habitat quality (Griffiths and Christian 1996).

A particularly important effect of disturbance on habitat structure for reptiles is the reduction in vegetation cover that can directly influence insolation levels and thereby alter microclimate conditions (Elzer et al. 2013; Lopera et al. 2022). For example, burned areas in the Great Smoky Mountains National Park, USA, provided microclimates that allowed for longer daily activity for the northern fence lizard ( *Sceloporus undulatus hyacinthinus* ) compared to unburned areas, leading to increased fitness and abundance of this species (Fouts et al. 2017). In Morocco, fires reduced tree cover in pine plantations, increasing thermoregulation opportunities and thus positively influencing reptile abundance (Chergui, Fahd, and Santos 2019).

In addition to microclimate, food resources are a key component of habitat quality that is affected by disturbance (Pianka and Goodyear 2012; Wijas, Finlayson, and Letnic 2023). For example, insectivorous geckos displayed increased body condition and positive population growth in recently burned areas due to an increased abundance of invertebrate prey (Smith 2018). In South Australia's arid region, kangaroo grazing reduced vegetation cover, leading to a decline in termite abundance and subsequently termite‐eating lizards (Wijas, Finlayson, and Letnic 2023).

Predation risk and competitive interactions are other important components of habitat quality for terrestrial reptiles that can be altered by disturbance through habitat simplification (Agriculture: Anderson and Burgin 2008; Hansen et al. 2019; Mowing: Sato et al. 2014; Fire: Wilgers and Horne 2007). For example, for large snakes in the tallgrass prairie of North America, the lack of cover following a fire increases predation risks from aerial predators (Wilgers and Horne 2007). Similarly, Law et al. (2020) found that birds more frequently attacked clay models of common European adders ( *Vipera berus* ) in areas grazed by sheep. However, following prescribed burning there was no significant change in predator activity at burrows of the Australian great desert skink (*Liopholis kintorei*) (Moore et al. 2018). The activity of great desert skinks and their predators were also not influenced by longer term effects of fire on vegetation cover, although skinks spent more time fully outside their burrow entrances where vegetation cover was higher (Ridley and Schlesinger 2023). Although competitive interactions are less studied, there is evidence that habitat simplification due to grazing reduced competition of geckos in an Australian tropical savanna, which benefits generalist species (Nordberg and Schwarzkopf 2019).

## 4 Principle 3. Species Responses to Disturbance Are, to a Large Degree, Determined by Their Responses to Habitat Openness

Our structured review strongly supports the notion that disturbance‐driven reduction in vegetation cover, especially understorey vegetation, is a key mechanism by which disturbance affects reptile communities (Figure 4; Appendix 2: Table A1), and this is reflected in the broader literature (Daly, Dickman, and Crowther 2008; Elzer et al. 2013; Fouts et al. 2017). Species‐specific preferences for different levels of habitat openness (Daly, Dickman, and Crowther 2008) mean that changes in habitat openness caused by disturbance often result in predictable patterns of ‘winner–loser’ replacement in reptile communities, as is the case for ants. Disturbance typically benefits taxa that prefer open habitats, such as arid‐adapted species (Trainor and Woinarski 1994) or are adaptable to them (generalist species; Masters 1996; Taylor et al. 2020). Conversely, disturbance disadvantages taxa that prefer closed habitats, such as forest specialists (Akani et al. 2018) and other shade‐tolerant species (Pike, Webb, and Shine 2011). For example, prescribed burning in Brazilian savannas negatively affects lizard species such as the black‐spotted skink *Copeoglossum nigropunctatum* that prefer more humid habitats, and favour those that prefer more open habitats, such as the lizards *Tropidurus itambere* and *Micrablepharus atticolus* (Costa et al. 2020). Conversely, when introduced plants increase cover in previously open habitats, reptiles adapted to open environments are detrimentally impacted (Schlesinger et al. 2020). In North American sand‐pine scrub, high‐intensity burning promotes arid‐adapted species, such as the six‐lined racerunner ( *Aspidoscelis sexlineatus* ) and the Florida scrub lizard (*Scleropus woodi*), but disadvantages species that prefer more moist habitats, such as the southeastern five‐lined skink (*Plestiodon inexpectatus*) (Greenberg, Neary, and Harris 1994). Similarly, slash‐and‐burn practices in Nigerian tropical forest eliminated forest specialist species of chameleon (*Chameleo*) and snakes (e.g., *Bitis*, *Naja*), but it promoted species such as the common agama ( *Agama agama* ) that prefer open habitats (Akani et al. 2018).

Increasing habitat openness also tends to benefit reptiles with ambush foraging modes (Griffiths and Christian 1996), whereas actively foraging reptiles may be disadvantaged, particularly smaller species that rely on vegetation for shelter while foraging. For example, wildfire in Australia's Morton National Park reduced the survival of the actively foraging small‐eyed snake ( *Cryptophis nigrescens* ), but not that of the broad‐headed snake ( *Hoplocephalus bungaroides* ), an ambush predator (Webb and Shine 2010). Following disturbance in more open habitats, actively foraging reptiles may face higher predation risks (Huey and Pianka 1981; Pianka 2017) and greater microclimatic challenges (Huey 1991).

Species‐specific responses to disturbance‐mediated habitat openness scale up to predictable effects of disturbance at the community level. For example, in North America, long‐term prescribed burning in fire‐suppressed longleaf pine forests reshaped reptile assemblages to be more similar to those in the reference sites with lower vegetation cover (Steen et al. 2013). This change in community composition was driven mainly by the decline of hardwood forest species that prefer more humid habitats, and the increase of open scrub species (Steen et al. 2013). In tropical northern Australia, the replacement of native forests with shadier pine forests resulted in distinct reptile assemblages associated with closed habitats (Mott, Alford, and Schwarzkopf 2010). In the Great Victoria Desert of Western Australia, Pianka and Goodyear (2012) observed a turnover of dominant species in the lizard assemblages over a 16‐year fire succession cycle. The generalist central netted dragon ( *Ctenophorus nuchalis* ) was numerically dominant after fires, but as time since fire increased and vegetation cover increased, it was progressively replaced by the central military dragon (*Ctenophorus isolepsis*), which prefers shadier habitats. Similarly, repeated wildfires in southern France caused a shift in dominant species from those associated with forest habitats to species that prefer open habitats (Santos and Cheylan 2013; Santos et al. 2019). The reverse pattern of a shift in dominant species occurred in sand‐mined forests in eastern Australia as vegetation cover gradually recovered following the cessation of mining (Taylor and Fox 2001).

Other community metrics, such as diversity and richness, can also be predicted by species‐specific responses to disturbance‐mediated habitat openness. However, the direction of changes in these metrics depends on how disturbance‐altered habitat openness influences the state of habitat conditions and the occurrence of species replacement. For example, canopy removal in the Gum Plateau of southeastern Australia increased species richness by creating more heterogeneous habitat, allowing open habitat specialists to coexist with closed habitat specialists (Pike, Webb, and Shine 2011). In contrast, reduced vegetation cover in temperate grasslands and woodlands of southeastern Australia led to decreased species richness and diversity due to habitat simplification (Howland et al. 2014).

Contrary to the examples above, dramatic changes in vegetation cover due to high‐intensity wildfires did not affect lizard abundance in a southern United States mixed pine‐hardwood forest (Duarte, Brown, and Forstner 2017). Similarly, there were no detectable changes in reptile species composition due to hurricane‐mediated changes of vegetation structure in the Virgin Islands (Richter et al. 2021). These limited impacts indicate that habitat conditions remained suitable despite the changes in vegetation structure (Duarte, Brown, and Forstner 2017; Richter et al. 2021).

Our structured review identified only five (out of 71) studies where factors other than vegetation change were identified as drivers of reptile responses. Two of the studies identified reduced food resources as the key factor (Nordberg et al. 2018; Wijas, Finlayson, and Letnic 2023), although this might have been caused by changed vegetation structure (Daly, Dickman, and Crowther 2008; Wijas, Finlayson, and Letnic 2023). Overall, however, the proposition that species responses to disturbance are, to a large degree, determined by their responses to habitat openness, is highly applicable to terrestrial reptiles.

## 5 Principle 4. The Same Disturbance Will Have Different Effects on Communities in Different Habitats, Because of Different Impacts on Habitat Openness

This principle proposes that the same disturbance will have a greater impact on communities in closed than open habitats because it results in greater changes in habitat openness (Andersen 2019). Species responses to disturbance are therefore habitat‐dependent, varying according to change in habitat favourability relating to habitat openness (Andersen 2019). The applicability of this principle to terrestrial reptiles is supported by almost all relevant studies (10 out of 11 studies) in our structured review (Figure 4; Appendix 2: Table A1), and by the broader literature.

Although not statistically significant, a recent meta‐analysis of reptile responses to anthropogenic disturbance found that the greatest negative effect size was in forest and the least was in grassland (Doherty et al. 2020). Results of meta‐analyses are typically confounded by a high level of uncontrolled variation in the type and intensity of disturbance, which can mask predictable patterns of response (Andersen 2021). Individual studies show stronger support for the proposition that the strength of reptile responses to disturbance varies predictably with vegetation type and habitat structure, and specifically in relation to relative changes in habitat openness (Chergui, Fahd, and Santos 2019; Hu et al. 2016; Letnic et al. 2004; Lindenmayer et al. 2008; Mott, Alford, and Schwarzkopf 2010; Rochester et al. 2010; Rotem et al. 2016). For example, wildfire in southern California had a significant impact on the diversity and composition of reptiles in chaparral and coastal sage scrub habitats, but not in grasslands; reduction and recovery time of vegetation cover was far greater in the structurally complex habitats of chaparral and coastal sage scrub than in grasslands (Rochester et al. 2010). In north‐western Africa, wildfire affected the abundance and functional richness of reptiles in pine forests, but not in cork oak forests due to more persistent vegetation structure change in the former habitat (Chergui, Fahd, and Santos 2019; Chergui et al. 2020). Similarly, reptiles showed contrasting responses to grazing in mesic versus arid habitats of Israel (Rotem et al. 2016) and to timber harvesting in wet versus lowland forests of southeast Australia due to differences in habitat openness (Alexander, Scotts, and Loyn 2002). The one study (out of 11) that did not support the principle is where consecutive hurricanes resulted in no significant changes in the composition of reptile communities of different habitats in the US Virgin Islands (Richter et al. 2021).

Responses of individual species to disturbance can vary in different habitats because of different effects on habitat openness, and patterns of community succession can vary because optimal habitat openness occurs at different times since disturbance in different habitats (Alexander, Scotts, and Loyn 2002; Letnic et al. 2004; Santos and Poquet 2010; Taylor and Fox 2001). Additionally, for a single species, the optimal habitat openness can vary across environments (Frishkoff et al. 2019). For example, in North America, the xeric‐adapted six‐lined racerunner ( *Aspidoscelis sexlineatus* ) was most abundant recently after fire in sandhill and sand pine scrub habitats (Greenberg, Neary, and Harris 1994; Mushinsky 1985), whereas in rosemary scrub it was most abundant in areas with intermediate fire ages (Ashton and Knipps 2011). Similarly, in southeastern Australia, the abundance of the yellow‐bellied water skink (*Eulamprus heatwolei*) increased with time since fire in coastal banksia woodland but decreased in lowland eucalypt forest (Hu et al. 2016).

## 6 Principle 5. Community Responses to the Same Disturbance Will Vary According to Functional Composition and Biogeographic History in Relation to Habitat Openness

This principle proposes that faunal responses to disturbance are influenced by their contemporary and historical biogeography in relation to habitat openness. Taxa whose distributions and diversity are more strongly concentrated in more arid regions (and therefore more open habitats), or belong to lineages with longer evolutionary histories in more arid environments, are likely to be favoured by moderate disturbance at the mesic (less open) ends of their ranges (Andersen 2019). Conversely, mesic‐adapted species at the same sites are likely to be disadvantaged by disturbance.

There is strong evidence that reptiles follow this principle, supported by all six relevant studies from the structured review (Appendix 2: Table A1) along with the broader literature. For example, reptiles in the transition zone between Mediterranean and Atlantic bioregions in northern Portugal display contrasting responses to fire based on their biogeographic affinities (Ferreira, Mateus, and Santos 2016). These bioregions have functionally different reptile communities: Mediterranean species typically prefer warm environments that are provided by open habitats, whereas Atlantic species typically prefer cool, moist environments that are provided by closed canopy habitats (Sillero et al. 2009). As such, Mediterranean species respond positively to fire, while Atlantic reptile species respond negatively (Ferreira, Mateus, and Santos 2016). Such contrasting responses to fire have also been observed in the transition zone between Mediterranean and medio‐European reptile species in southeastern France, where medio‐European reptiles associated with forest habitats respond negatively to fire, and Mediterranean species respond positively (Santos et al. 2019). Similarly, in a study of grazing impacts in the Israeli arid zone, reptile species with arid biogeographic affinities responded positively, whereas Mediterranean species were negatively affected (Rotem et al. 2016). The opposite response occurred to afforestation that increased vegetation cover in the Israeli arid zone, where arid‐adapted species were negatively impacted (Hawlena and Bouskila 2006; Hawlena et al. 2010) and Mediterranean species were positively impacted (Hawlena and Bouskila 2006) by the alteration of habitat openness.

## 7 Discussion

Our synthesis has revealed that, to a large extent, responses of terrestrial reptiles to disturbance conform with the five principles previously proposed for understanding the global disturbance dynamics of ant communities (Andersen 2019). Our structured review covered a broad range of biomes from all continents where reptiles occur, as well as covering an extensive range of disturbance agents. Lizards were the most‐studied reptile group and turtles the least, as reflects their relative abundance and diversity. This representation of studies is sufficient for us to conclude that, as has been shown for ants: (1) the impact of disturbance on, and functional composition of species in relation to, habitat openness are fundamental for understanding reptile responses to disturbance; (2) reptile survival during or immediately following disturbance seems to be typically high, whereas the indirect effects of disturbance on them, especially through habitat modification, are well established; (3) variation among reptile species in their responses to a disturbance is strongly influenced by their preference for habitat openness; (4) the same disturbance will have different effects on reptiles in different habitats, because of different impacts on habitat openness; and (5) historical and contemporary biogeography influence responses of terrestrial reptiles to disturbance by shaping functional composition of terrestrial reptiles in relation to habitat openness. To better understand the generality of these five principles, we suggest prioritising future studies in biomes within Asia and Africa, as these regions are underrepresented compared to others.

For ectotherms, a key impact of disturbance‐induced increases in habitat openness is the alteration of insolation and thereby microclimate conditions, particularly temperature and humidity. Although ectothermic species can compensate for immediate changes in the thermal and moisture environment by seeking micro‐climatic refugia or through changes in body posture, the altered microclimate caused by increased habitat openness is likely to have an important impact on fitness (Vickers, Manicom, and Schwarzkopf 2011). Ecophysiological traits relating to temperature and moisture are therefore likely to have an especially important influence on reptile responses to disturbance. Physiological traits of terrestrial reptiles play a key role in coping with changes in the thermal environment (Ferreira, Santos, and Carretero 2016; Nowakowski et al. 2018) and species that prefer open or warm habitats typically have higher thermal tolerance than do forest‐adapted or generalist species (Brusch, Taylor, and Whitfield 2016). Similarly, sun‐seeking species have higher thermal tolerance compared to sun‐avoiding species in the same habitat (Muñoz et al. 2016). The same pattern also occurs for populations within species, where populations that live in open habitats have higher thermal tolerance than populations that live in closed habitats (Yuan et al. 2018). Species or populations with high thermal tolerance are less sensitive to habitat modification than species or populations with low thermal tolerance (Nowakowski et al. 2018), which provides a mechanistic basis for habitat openness‐mediated variation among species in their responses to disturbance.

Morphological traits also play a critical role in the ability of species to utilise habitats and to persist when habitat conditions are altered (Miles 2020). For example, the length of limbs in lizards affects their locomotor performance (Losos 1990), which is important for foraging success and predator escape (Goodman 2007; Melville and Swain 2000; Pianka 1969). The longer legs of many lizards living in open habitats allow for greater sprinting speed (Melville and Swain 2000), while the shorter legs of many lizards living in closed, dense habitats provide greater stability and manoeuvrability for efficient movement (Goodman 2007; Melville and Swain 2000; Losos 1990). Another example is body colouration, which influences thermoregulation performance of reptiles (Jin, Tong, and Zhang 2016; Sun et al. 2024) and may mediate how well reptiles can adapt to microclimatic fluctuations. Darker colouration is more beneficial for species that live in closed habitats than in open habitats, as it more effectively absorbs heat from limited solar radiation (Jin, Tong, and Zhang 2016; Sun et al. 2024). Thus, species with morphological traits well‐suited to post‐disturbance habitat conditions can more effectively exploit resources, maintaining their fitness for survival and persistence.

Some species traits can change in response to disturbance‐mediated environmental change, including alterations in habitat openness (Crispo et al. 2010; Lyndon‐Gee and Jessop 2020; Wild and Gienger 2018). Such trait adaptation may be rapid, such as physiological or behavioural change in response to recent disturbance, or may occur over generational timescales, such as with morphological traits (Crispo et al. 2010; Lyndon‐Gee and Jessop 2020). For example, fire‐mediated changes in habitat openness that increased the range of environmental temperatures, induced rapid physiological changes that enhanced locomotor performance in the eastern fence lizard ( *Sceloporus undulatus* ) (Wild and Gienger 2018).

Given the relevance of habitat openness to so many functional traits, the five principles developed by Andersen (2019) can be expected to apply to the disturbance dynamics of many animal groups and not just ectotherms. The published literature suggests that they are indeed more broadly applicable. A recent systematic review of tetrapod mortality during fire found that, on average, only 3% of animals were directly killed (Jolly et al. 2022). This strongly supports Principle 1, that the most important effects of disturbance on fauna are typically indirect through habitat modification, at least in relation to fire. Principle 1 appears to hold very broadly across taxa for faunal responses to fire in Australian tropical savannas (Andersen 2021). Habitat openness is also an important factor influencing variation in species composition (Principle 2) in both birds (Montano‐Centellas and Garitano‐Zavala 2015; Rodrigues et al. 2016) and mammals (Burns et al. 2011; Coppeto et al. 2006). Habitat openness mediates the responses of birds and mammals to disturbance (Principle 3). For example, in Brazilian savanna, increased canopy openness after fire promoted small mammal species from more open savanna habitats at the expense of forest specialist species (Camargo et al. 2018). Similarly, in the tropical Andes, generalist bird species were more resilient than forest‐dependent species to increased habitat openness arising from anthropogenic disturbance (Montano‐Centellas and Garitano‐Zavala 2015). The reverse pattern was observed in northern Australia, where long‐term savanna fire exclusion led to a dramatic reduction in vegetation openness and an increase in abundance of forest‐specialist birds and mammals at the expense of savanna specialists (Woinarski, Risler, and Kean 2004).

There are many examples among ectothermic taxa other than ants and reptiles of habitat type influencing faunal responses to disturbance that can be linked to differences in habitat openness (Principle 4). For example, the change in functional composition of bees after fire was more marked in a European temperate (higher rainfall) compared with Mediterranean region (Moretti et al. 2009). In southern California, the effect of fire on the western toad ( *Anaxyrus boreas* ) was stronger in structurally complex than in structurally simple habitats (Rochester et al. 2010). However, there are few relevant studies for birds or mammals. Finally, at least for birds, faunal responses to disturbance are influenced by their biogeography in relation to habitat openness (Principle 5). In Mexico, for example, Nearctic passerine birds were more resilient to increasing habitat openness due to urbanisation than were Neotropical species (González‐Oreja 2011).

In conclusion, a framework based on habitat openness is highly applicable to terrestrial reptiles and appears to have wide applicability to a predictive understanding of faunal responses to disturbance more generally. Such an understanding is based, to a large extent, on phenomenological research that describes patterns of responses. We suggest that a priority for future research is to improve our mechanistic understanding of disturbance responses through a focus on the functional traits underlying them. Multiple traits may interplay to influence how animals from different trophic levels respond at different times and spatial scales following disturbance (Ensbey et al. 2023; Schleuning, García, and Tobias 2023). For ectotherms such as ants and reptiles, physiological traits related to temperature and moisture are likely to be a key factor influencing disturbance responses (Kearney and Porter 2009). There is a priority need for field and laboratory experiments that test the importance of ecophysiology and other potential factors such as behaviour, morphometry, food resources and predation as functional drivers of faunal responses (Daly, Dickman, and Crowther 2008). For reptiles, for example, measurements of microclimate and body temperature could be taken along gradients of habitat openness in the field, with these complemented by laboratory experiments on physiological traits related to temperature and humidity and their plasticity, which can strengthen our inference. Such mechanistic understandings will substantially enhance our ability to predict the responses of faunal communities to shifts in disturbance regimes.

## Author Contributions

**Angga Rachmansah:** conceptualization (equal), data curation (equal), formal analysis (equal), writing – original draft (equal), writing – review and editing (equal). **Keith Christian:** writing – original draft (equal), writing – review and editing (equal). **Brett P. Murphy:** writing – original draft (equal), writing – review and editing (equal). **Christine Schlesinger:** writing – original draft (equal), writing – review and editing (equal). **Alan N. Andersen:** conceptualization (equal), writing – original draft (equal), writing – review and editing (equal).

## Conflicts of Interest

The authors declare no conflicts of interest.

## Data Availability

The authors have nothing to report.

## Appendix 2

**TABLE A1:** Studies of reptile responses to disturbance included the structured review of relevant papers published between 2017 and 2022 inclusive (including early access). Their findings are summarised in relation to four principles identified for ants (Andersen 2019): (1) The most important effects of habitat disturbance are typically indirect, via habitat structure, microclimate, resource availability and competitive interactions; (3) Species responses to disturbance are to a large degree determined by their responses to habitat openness; (4) The same disturbance will have different effects in different habitats, because of different impacts on habitat openness; (5) Community responses to the same disturbance will vary according to functional composition and biogeographic history in relation to habitat openness. Studies supporting and contradicting the principles are highlighted in green and orange respectively.

Study	Taxa	Disturbance	Region	Biome	Metric	Principle 1	Principle 3	Principle 4	Principle 5
Senior et al. (2023)	Reptile (lizard and snake)	Prescribed fire	Australia	Mediterranean woodland	Abundance, richness	Importance of post‐fire environment noted	Responses related to amount of unburnt vegetation within a site		
Webb et al. (2023)	Snake ( *Hoplocephalus bungaroides* )	Natural fire	Australia	Temperate forest	Abundance, occupancy	Higher occupancy rates following fire indicated low mortality; responses related to changes in vegetation structure	Responses related to occurrence of open sunny sites that were previously shaded		
Aviles‐Rodriguez, De León, and Revell (2023)	Lizard ( *Anolis cristatellus* )	Hurricane	Central America	Tropical forest	Composition, density	Responses related to changes in vegetation structure	Responses due to canopy defoliation along with other factors		
Silva et al. (2022)	Lizard	Fire, pasture	South America	Tropical forest	Abundance, richness	Responses related to changes in vegetation structure	Responses related to the structural complexity of the surrounding site: the abundance of sun‐avoiding lizards increased in sites with more complex structure (shaded environment), and decreased in the sites that were significantly affected by fire		
Wijas, Finlayson, and Letnic (2023)	Mammal and reptile (lizard)	Grazing	Australia	Xeric shrubland	Abundance	Disturbance reduced vegetation cover and abundance of prey	Responses related to food resources		
Royal et al. (2022)	Reptile (lizard and snake)	Forest management (fire, harvest, etc.)	North America	Temperate forest	Occupancy, richness	Responses related to changes in vegetation structure	The occupancy probability of most open‐pine‐associated species was positively related to open canopy conditions, whereas the occupancy probability of mesic‐forest‐associated species was positively related to closed canopy conditions		
de Jesús Cervantes‐López et al. (2022)	Amphibian and reptile (lizard and snake)	Agroforestry management (harvest, etc.)	Central America	Tropical forest	Abundance, richness, composition	Responses related to changes in vegetation structure	Responses related to relative humidity of microhabitats, and therefore level of openness		
Santos et al. (2022)	Reptile (lizard and snake)	Natural fire	Europe	Mediterranean scrub	Abundance, richness, composition	Results indicate in situ persistence	Responses related to vegetation recovery and thermal‐moisture environment		Mediterranean species (with an evolutionary history with fires and favour open habitats) associated with higher fire recurrence
Heaton et al. (2022)	Amphibian and reptile	Prescribed fire	North America	Temperate forest	Abundance, richness, composition	Significant turtle mortality indicated	Responses related to both habitat structure and food availability		
Lazzari, Sato, and Driscoll (2022)	Reptile (lizard and snake)	Prescribed fire	Australia	Mediterranean woodland	Abundance, richness	Both mortality and habitat change suggested as explanations	Habitat structure change and mortality suggested as explanations		
Wong et al. (2021)	Pink‐tailed worm lizard ( *Aprasia parapulchella* )	Grazing, agricultural modification	Australia	Temperate grassland	Abundance	Responses related to changes in vegetation structure	Responses related to changes in grass cover		
Infante et al. (2021)	Lizard	Natural fire	South America	Temperate forest	Density, richness	Responses related to changes in vegetation structure	Responses related to vegetation cover		
Muñoz, Felicísimo, and Santos (2021)	Reptile (lizard and snake)	Natural fire	Europe	Mediterranean scrub	Abundance, richness, composition	Responses related to changes in vegetation structure	Noted the importance of reduction in tree cover, with heliothermic species displaying positive responses to fire	Vegetation type influenced responses, with sites that had slower vegetation recovery had a more significant changes in reptile assemblage	
Neilly, Ward, and Cale (2021)	Mammal and reptile (lizard)	Grazing (destocking/passive restoration)	Australia	Mediterranean woodland	Abundance, richness, composition	Responses related to changes in vegetation structure	Responses related to changes in vegetation cover	Responses differed between vegetation types due to unique vegetation recovery trajectories	
Marroquín‐Páramo et al. (2021)	Amphibian and reptile (lizard and snake)	Hurricane	Central America	Tropical forest	Abundance, richness, composition	Responses related to changes in vegetation structure	Responses presumably related to changes in vegetation structure and food resources	Changes in lizard abundance were more apparent in old‐growth forests than in other vegetation types	
Cabrera (2021)	Lizard	Tree removal, soil compaction	South America	Xeric shrubland	Abundance	Responses related to changes in vegetation structure	Responses related to changes in vegetation cover and soil conditions		
Terán‐Juárez et al. (2021)	Lizard	Various anthropogenic disturbance (logging, agriculture)	Central America	Tropical forest	Density, occupancy	Responses related to changes in vegetation structure	Responses related to changes in vegetation cover		
Richter et al. (2021)	Amphibian and reptile (lizard and snake)	Hurricane	Central America	Tropical forest	Diversity	No detectable changes in a short time (< 1 year) after hurricane, it may indicate high survival	Vegetation structure changes indicated; no alteration in reptile community presumably due to their resistance and adaptability to recover	Pre and post hurricanes, diversity and richness did not differ significantly in all the vegetation types	
Yudha et al. (2021)	Reptile (lizard and snake)	Logging	Asia	Tropical forest	Abundance	Responses related to changes in vegetation structure	Responses related to age stands, with higher abundance in younger forest ages presumably due to greater canopy openness		
Buchanan, Steeves, and Karraker (2021)	Turtle	Prescribed fire	North America	Temperate forest	Mortality	Significant turtle mortality (47 across 31.2 ha)			
Souza et al. (2021)	Lizard	Natural fire	South America	Tropical savanna	Density	Responses related to changes in vegetation structure	Responses related to changes in vegetation cover and other unmeasured factors		Lizards in (open) savannas are not found in the adjacent (closed) forests, and they displayed high a resilience to fire
Howze and Smith (2021)	Snake	Prescribed fire	North America	Temperate forest	Occupancy	Responses related to changes in vegetation structure	Responses related to open‐canopy forest structure. Species of open‐canopied forest specialists occurred in sites within a narrower range of fire intervals (1.4–2.5 years) than generalist species (2–10 years)		
Jordaan et al. (2020)	Reptile (lizard and snake)	Prescribed fire	Africa	Tropical savanna	Mortality	Mortality ranged from 2.3 to 46.3 ha^−1^, depending on fire			
Jones et al. (2020)	Amphibians and reptiles (lizard)	Forest management (harvest, thinning, short rotation harvest)	North America	Temperate forest	Occupancy	Responses related to changes in vegetation structure	Species responses attributed to different openings patches and structure availability resulting from each forest management treatment		
Badillo‐Saldaña, Castellanos, and Ramírez‐Bautista (2020)	Lizard	Farming management	Central America	Tropical forest	Abundance, richness, diversity	Responses related to changes in vegetation structure	Responses were possibly linked to habitat openness through modification thermal of conditions		
Costa et al. (2020)	Lizard	Prescribed fire	South America	Tropical savanna	Abundance, composition	Responses related to changes in vegetation structure	Responses due to changes in habitat structure that influenced microclimate conditions. Species that prefer open habitats, favoured burnt plots, whereas species that prefer more mesic habitats, favoured unburnt plots		
Chergui et al. (2020)	Reptile	Natural fire	Africa	Mediterranean forest	Richness	Noted the importance of post‐fire environments	Contrasting responses of species to fire related to open vs. complex microhabitats	Responses depended on forest type due to different impacts on habitat openness and structure	Reptile community in cork oak forests was mainly composed of small Mediterranean lizards that possess functional traits that are well adapted to fire prone areas (open habitats)
Durigan et al. (2020)	Multiple taxa, including reptile (lizard)	Prescribed fire	South America	Tropical savanna	Abundance, richness, composition	Responses related to changes in vegetation structure	Responses related to changes in vegetation structure (tree density and basal area)		A high percentage of open habitat specialist species in the species composition of the study sites resulted in negligible impacts of fire
Jiménez‐Albarral, Pleguezuelos, and Santos (2020)	Reptile (lizard and snake)	Windstorms	Europe	Mediterranean forest	Density, richness	Responses related to changes in vegetation structure	Responses related to reduction in tree density and canopy		
Bateman and Riddle (2020)	Lizard (*Aspidocelis*, *Scleropus*)	Natural fire	North America	Xeric shrubland	Abundance	Both direct mortality and habitat changes suggested as explanations	Responses attributed to types of foraging activity and habitat changes; Active foragers at a greater risk of delayed mortality (high temperature)		
Schlesinger et al. (2020)	Reptile (lizard and snake)	Restoration (weed removal)	Australia	Xeric shrubland	Abundance, richness, composition	Responses related to changes in vegetation structure	Responses related to changes in ground cover		
Taylor et al. (2020)	Bird and reptile (lizard and snake)	Natural fire	Australia	Temperate woodland	Abundance, richness	Responses related to changes in vegetation structure; shelter provided by rocky outcrops likely reduced mortality during fire	Responses related to impacts on vegetation structure		
Graitson, Ursenbacher, and Lourdais (2020)	Snake ( *Coronella austriaca* )	Management interventions (grazing, mowing, control)	Europe	Temperate grassland	Abundance	Responses attributed to changes in vegetation structure and microclimate, food resources, and predation risks	Responses attributed to changes in the heterogeneity of vegetation structure and microclimate, food resources and predation risks		
Evans, Newport, and Manning (2019)	Reptile	Restoration (CWD addition, grazing, prescribed fire)	Australia	Temperate woodland	Abundance, richness	Responses related to changes in vegetation structure	Responses attributed to changes in vegetation cover	Responses to restoration management depended on the vegetation cover condition of the sites	
Chergui, Fahd, and Santos (2019)	Reptile	Natural fire	Africa	Mediterranean forest	Abundance, richness	Responses related to changes in vegetation structure	Responses related to change in tree cover and bare soil	Responses depended on vegetation type due to the reduction of impacts of fire on habitat openness (e.g., tree cover, bare soil) in cork oak forest than in pine forest	
Simms et al. (2019)	Lizard	Natural fire	Australia	Xeric shrubland	Occupancy	Responses related to changes in vegetation structure	Responses related to local variation in habitat openness		
Scroggie et al. (2019)	Stripped legless Lizard ( *Delma impar* )	Natural Fire, grazing	Australia	Temperate grassland	Occupancy	Responses related to changes in vegetation structure	Responses attributed to change in vegetation structure		
Crowley and Preece (2019)	Lizard (gecko, skink)	Flood	Australia	Tropical savanna	Composition	Suggested direct mortality may have had a more significant impact than habitat modification based on the lack of understorey reptiles after the flood	Responses related to reduction of shrub abundance		
Fernandes et al. (2019)	Lizard (*Psammodromus algirus*)	Grazing	Europe	Mediterranean woodland	Abundance	Responses related to changes in vegetation structure	Responses related to vegetation structure and level of insolation		
Kellner et al. (2019)	Mutiple taxa, including reptile	Logging	North America	Temperate forest	Abundance, richness	Responses related to changes in vegetation structure	Responses related to open microhabitats		
Halstead et al. (2019)	Snake ( *Thamnophis sirtalis tetrataenia* )	Prescribed fire	North America	Mediterranean scrub	Survival	No carcasses were found after the fire. Changes in habitat structure were likely to have a stronger impact	Responses related to changes in vegetation structure		
Oliveira et al. (2019)	Turtle (*Rhinoclemmys* sp.)	Natural fire, drought	South America	Tropical forest	Mortality	48% direct mortality during fire			
Bakiev, Gorelov, and Klenina (2019)	Sand lizard (*Lacerta agilis*)	Natural fire	Europe	Temperate grassland	Abundance	Responses related to changes in vegetation structure	Responses presumably related to changes in vegetation cover		
Santos et al. (2019)	Amphibian and reptile	Natural fire	Europe	Mediterranean forest	Abundance	Responses related to changes in vegetation structure	Responses related to changes in habitat openness		Most Mediterranean reptile species benefited from fire as they prefer open habitats, whereas medio‐European reptile species disadvantaged from fire as they prefer forested habitats
Val et al. (2019)	Reptile	Grazing	Australia	Xeric shrubland	Richness	Noted direct effects of grazing, presumably due to trampling or behaviour alteration, were stronger than habitat modification (woody cover, native plant richness)	Responses related to changes in woody cover		
Nordberg and Schwarzkopf (2019)	Gecko ( *Gehyra dubia* , *Oedura castelnaui* , *Strophurus williamsi* )	Grazing	Australia	Tropical savanna	Abundance, composition	Noted the importance of reduced competition due to habitat homogenisation and simplification	Responses related to simplification of habitat structure		
Moreno Rueda et al. (2019)	Lizard (*Psammodromus algirus*)	Natural fire	Europe	Mediterranean scrub	Abundance	A crash in abundance indicated significant mortality, but responses related to vegetation cover	Responses related to reduction in vegetation cover		
Greenberg, Zarnoch, and Austin (2019)	Reptile (lizard and snake)	Prescribed fire	North America	Temperate forest	Diversity, richness, evenness	Noted no or little evidence of direct mortality	Responses attributed to surface activity of reptiles unrelated to burns and changes in vegetation structure		
Nordberg et al. (2018)	Lizard ( *Gehyra dubia* , Cryptoblepharus australis)	Grazing	Australia	Tropical savanna	Abundance	Responses related to changed food resources	Responses related to food resources		
Neilly, Nordberg, et al. (2018)	Lizard	Grazing	Australia	Tropical savanna	Abundance, richness, composition	Responses related to changes in vegetation structure	Responses related to simplification of ground vegetation structure (grass cover, grass height, leaf litter, etc.)		
Suazo‐Ortuño, Urbina‐Cardona, et al. (2018)	Amphibian and reptile (lizard and snake)	Hurricane	Central America	Tropical forest	Abundance, richness, composition	Responses related to changes in vegetation structure	Responses attributed to changes in vegetation structure, influencing solar radiation, temperature, and humidty	Responses varied between successional stages of dry forest, with a more marked change in intermediate successional stages than in early and late successional stages	
Greenberg, Moorman, et al. (2018)	Amphibian and reptile (lizard)	Prescribed fire, mechanical vegetation removal	North America	Temperate forest	Abundance, richness	Responses related to changes in vegetation structure	Responses related to increased habitat openness		
Haby and Brandle (2018)	Reptile (lizard and snake)	Grazing exclusion	Australia	Xeric shrubland	Abundance, richness, composition	Responses related to changes in vegetation structure	Species‐specific responses varied depending on their preference for open or closed habitats	Responses varied between vegetation types due to different in vegetation recovery and other factors	
Neilly, O'Reagain, et al. (2018)	Reptile	Grazing	Australia	Tropical savanna	Abundance, richness	Responses related to changes in vegetation structure	Responses attributed to changes in vegetation structure		
Greenberg, Seiboldt, et al. (2018)	Amphibian and reptile	Prescribed fire	North America	Temperate forest	Abundance, richness	Responses related to changes in vegetation structure	Responses related to changes in canopy cover		
Suazo‐Ortuño, Benítez‐Malvido, et al. (2018)	Amphibian and reptile (lizard and snake)	Hurricane	Central America	Tropical forest	Abundance, richness, composition	Responses related to changes in vegetation structure	Responses presumably related to changes in vegetation structure, including reduced canopy cover	Responses varied between vegetation succession ages, where the abundance after the hurricane decreased in old‐growth forests, whereas it increased in the other vegetation succession ages (pasture, early, young and intermediate)	
Hromada et al. (2018)	Amphibian and reptile	Prescribed fire	North America	Temperate forest	Abundance, richness, composition	Responses related to changes in vegetation structure	Responses related to changes in vegetation cover		
Craig et al. (2018)	Reptile and mammal	Restoration	Australia	Mediterranean forest	Abundance, richness	Responses related to changes in vegetation structure	Responses related to changes in canopy cover		
Dixon et al. (2018)	Reptile	Natural and prescribed fires	Australia	Temperate forest	Abundance, richness, composition	Responses related to changes in vegetation structure	Responses related to changes in ground vegetation cover		
Gorissen, Greenlees, and Shine (2018)	Blue mountains water skink (*Eulamprus laurensis*)	Natural fire	Australia	Temperate forest	Abundance	Noted that high survival during fire and the importance of post‐fire habitat modification	Responses related to persistence of water (moisture) and changes in vegetation cover		
Pinto et al. (2018)	Lizard	Natural fire	Europe	Mediterranean scrub	Abundance, richness	Responses related to changes in vegetation structure	Responses related to tree cover	Noted evidence that vegetation types masked the responses of lizards to fire	Most of the lizards in the study area are Mediterranean species that prefer open habitats. Lizard evenness was lower in the long unburnt sites than in recent burnt sites
Pulsford et al. (2018)	Reptile	Grazing	Australia	Temperate woodland	Abundance, richness	Responses related to vegetation cover	Responses mostly related to vegetation cover		
Gorissen, Greenlees, and Shine (2017)	Blue mountains water skink (*Eulamprus laurensis*)	Longwall mining, anthropogenic disturbance	Australia	Temperate forest	Abundance	Responses related to changes in vegetation structure	Responses related to the persistence of water and changes in vegetation structure		
Lyndon‐Gee et al. (2017)	Lizard (*Eulamprus heatwolei*)	Logging	Australia	Temperate forest	Abundance	Responses related to changes in vegetation structure	Responses related to logging age and the extent of forest regeneration		
Carpio et al. (2017)	Lizard	Farming management	Europe	Mediterranean scrub	Abundance, richness, composition	Responses related to changes in vegetation structure	Species‐specific responses varied based on their preference for habitat openness		
Fouts et al. (2017)	Lizard ( *Sceloporus undulatus hyacinthinus* , *Plestiodon* spp.)	Prescribed fire	North America	Temperate forest	Abundance, occupancy	Responses related to changes in vegetation structure	Responses related to increased habitat openness and alteration in the thermal environment		
Rota et al. (2017)	Amphibian and reptile (lizard and snake)	Forest management (logging/harvest)	North America	Temperate forest	Abundance, richness	Responses related to changes in vegetation structure	Responses related to changes in habitat openness		
Lindenmayer et al. (2017)	Bird, mammal, and reptile	Weed management (spray and burn)	Australia	Temperate forest	Abundance, occupancy	Responses related to changes in vegetation structure	Responses related to vegetation removal and other factors		
Pulsford et al. (2017)	Reptile (lizard and snake)	Grazing	Australia	Temperate woodland	Abundance, richness	Responses related to changes in vegetation structure	Responses mostly related to vegetation cover		
Duarte, Brown, and Forstner (2017)	Lizard ( *Aspidoscelis sexlineata* , *Sceloporus consobrinus* , and *Scincella lateralis* )	Natural fire	North America	Temperate forest	Abundance	Low direct mortality indicated	No or little change of abundance of the studied lizard species in the short term despite marked dramatic changes to vegetation structure		
Parsons et al. (2017)	Reptile	Grazing	Australia	Tropical savanna	Abundance, richness	Responses related to changes in vegetation structure	Responses related to changes in the cover of vegetation		
Kay et al. (2017)	Reptile	Grazing	Australia	Temperate woodland	Abundance, richness, occupancy	Responses related to changes in vegetation structure	Responses attributed to changes in vegetation structural complexity		
Doherty et al. (2017)	Reptile	Natural fire	Australia	Mediterranean scrub	Abundance, richness, composition	Responses related to changes in vegetation structure	Responses related to fire age, reflecting species preferences to vegetation structure (canopy, litter, bare ground, etc.)		
Berriozabal‐Islas et al. (2017)	Lizard	Various anthropogenic disturbance	Central America	Tropical forest	Diversity, richness	Responses related to changes in vegetation structure	Responses attributed to changes in the structural complexity of vegetation		
